# Chromosome-level genome assembly of the threatened resource plant *Cinnamomum chago*

**DOI:** 10.1038/s41597-024-03293-1

**Published:** 2024-05-03

**Authors:** Lidan Tao, Shiwei Guo, Zizhu Xiong, Rengang Zhang, Weibang Sun

**Affiliations:** 1 Yunnan Key Laboratory for integrative conservation of Plant Species with extremely Small Populations, Kunming Institute of Botany, Chinese Academy of Sciences, Kunming, 650201 China; 2 CAS Key Laboratory for Plant Diversity and Biogeography of East Asia, Kunming Institute of Botany, Chinese Academy of Sciences, Kunming, 650201 China; 3 University of Chinese Academy of Sciences, Beijing, 101408 China; 4 Kunming Botanic Garden, Kunming Institute of Botany, Chinese Academy of Sciences, Kunming, 650201 China

**Keywords:** Plant genetics, Genome

## Abstract

*Cinnamomum chago* is a tree species endemic to Yunnan province, China, with potential economic value, phylogenetic importance, and conservation priority. We assembled the genome of *C. chago* using multiple sequencing technologies, resulting in a high-quality, chromosomal-level genome with annotation information. The assembled genome size is approximately 1.06 Gb, with a contig N50 length of 92.10 Mb. About 99.92% of the assembled sequences could be anchored to 12 pseudo-chromosomes, with only one gap, and 63.73% of the assembled genome consists of repeat sequences. In total, 30,497 genes were recognized according to annotation, including 28,681 protein-coding genes. This high-quality chromosome-level assembly and annotation of *C. chago* will assist us in the conservation and utilization of this valuable resource, while also providing crucial data for studying the evolutionary relationships within the *Cinnamomum* genus, offering opportunities for further research and exploration of its diverse applications.

## Background & Summary

The *Cinnamomum* genus (family: Lauraceae) comprises 248 species of evergreen trees or shrubs with a wide distribution spanning Tropical and Subtropical Asia to the Western Pacific^1^. *Cinnamomum* encompasses several economically important plant species that have versatile uses, including construction materials, furniture, spice production, pharmaceutical applications, and industrial oilseed purposes. Moreover, certain species from this genus, such as *C. camphora* and *C. japonicum*, are extensively cultivated as ornamental landscape trees^2,3^.

**Fig. 1:**
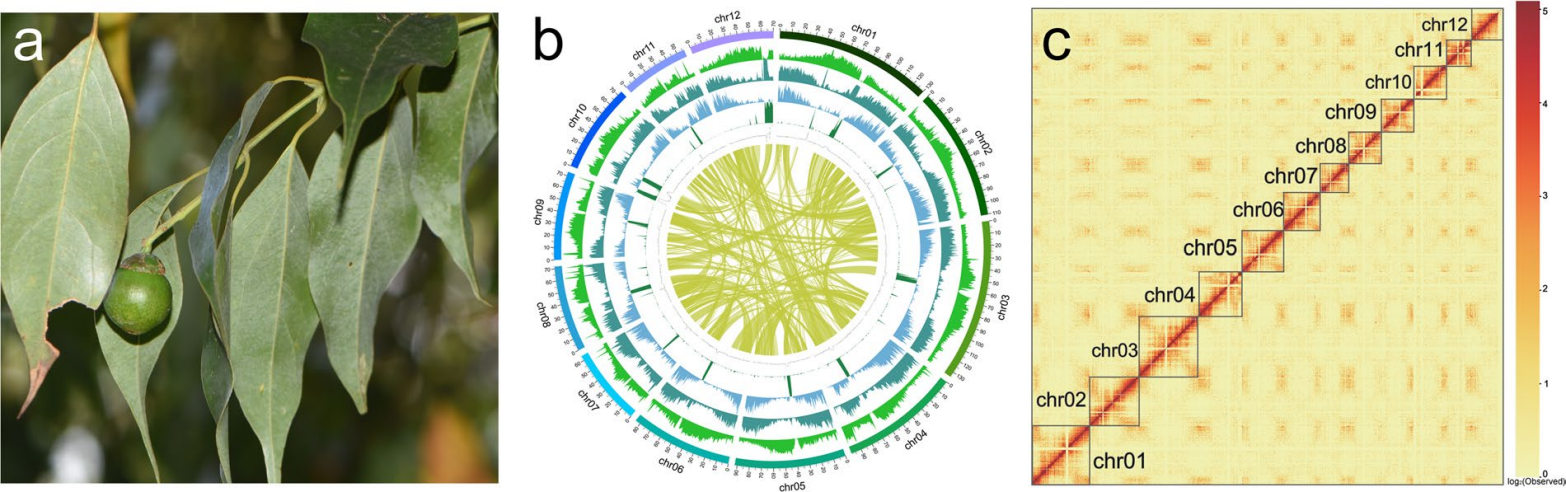
(**a**) Fruit and leaves of *Cinnamomum chago*. (**b**) The genome assembly of *C. chago* (window size: 500 kb). From outer to inner: chromosome coordinates, Class I TE density, Class II TE density, coding gene density, tandem repeat proportion, GC content, collinear blocks (minimum 100 kb). (**c**) Hi-C interactive heatmap (bin size = 100 kb).

*C. chago* B.S. Sun et H.L. Zhao is endemic to Yunnan province, China, and was initially discovered in La-Guo village, Yangbi county^4^ (Fig. 1a). Recent investigations have confirmed that *C. chago* is exclusively distributed in Dali Prefecture and Pu’er City of the province^5,6^. In Yunlong and Yangbi County of Dali Prefecture, mature seeds of *C. chago* were collected by villagers and sold by the local Yi people as traditional ethnic nut and traditional health products^5^. Preliminary nutritional analysis results revealed that *C. chago* seeds contain a high proportion of lauric acid indicating high potential for economic utilization^7^. Furthermore, the exceptional wood is frequently harvested for furniture production, significantly impacting its natural regeneration^6^.

Due to its small population size and intensive human disturbance, *C. chago* has been threatened and was assessed as one of the Plant Species with Extremely Small Populations (PSESP) in southwest China, requiring rescue protection in 2021^8,9^. Additionally, it was designated as one of China’s nationally protected Grade II wild plants, safeguarded by law. Moreover, its unique morphological features indicate that *C. chago* is a key phylogenetic taxon between the two sections of Asian *Cinnamomum* plants (Sect. *Camphora* and Sect. *Cinnamomum*)^5,10^. Therefore, a high-quality reference genome is crucial for promoting the conservation and utilization of *C. chago*, as well as studying the phylogeny of the family Lauraceae.

In this study, we assembled and annotated the genome of *C. chago* using PacBio HiFi reads (91.73 Gb, 80×), ONT reads (33.27 Gb, 30×), NGS reads (58.83 Gb, 50×), Hi-C reads (124.18 Gb), RNA-seq (16.31 Gb), and Iso-Seq (18.54 Gb). The assembled contig size was close to the estimated genome size of 1.1 Gb based on k-mer estimates, with a scaffold N50 length of 92.10 Mb. Approximately 99.92% of the assembled data were anchored onto 12 pseudo-chromosomes (Table 1; Fig. 1b,c; Supplementary Table S1). The chloroplast and mitochondrial genomes were 152,753 bp and 707,525 bp, respectively. A total of 1,366,885 repeat sequences were identified, with an approximate cumulative length of 676.3 Mb, accounting for 63.73% of the assembled genome. Of the identified repeats, long terminal repeats (LTRs) constituted the largest proportion, with a number of 466,655 and a cumulative length of 431,972,996 bp, accounting for 40.71% of the *C. chago* genome assembly. The genome contained 30,497 genes, including 28,681 protein-coding genes (Table 2). The high-quality reference genome and annotation information of *C. chago* will enhance our understanding of the evolutionary relationships within the genus *Cinnamomum*, and further research and utilization of the economically valuable resources.

**Table 1:** Summary of *Cinnamomum chago* genome assembly.

Parameter	Genome
Genome size	1,061,147,747 bp
GC content	39.36%
Contig number	15
Contig N10	134,349,014 bp
Contig N50	92,102,069 bp
Contig N90	56,592,361 bp
Scaffold number	14
Scaffold N10	134,349,014 bp
Scaffold N50	92,102,069 bp
Scaffold N90	64,088,380 bp
Gap number	1
Chromosome number	12
Chromosome length	1,060,287,469 bp (99.92%)
Mitochondria length	707,525 bp (0.07%)
Chloroplast length	152,753 bp (0.01%)

**Table 2:** Summary of *Cinnamomum chago* genome annotations.

Feature	Total Number	Coding Genes Number
gene	30,497	28,681
transcript	49,955	48,139
CDS	48,139	48,139
exon	307,936	306,097
intron	257,981	257,958

## Methods

### Sampling

For genomic DNA extraction, fresh young leaves of *C. chago* were collected from a single adult plant in Xincun village, Yangbi County, Dali Prefecture, Yunnan Province, China (25°33′37″N, 99°55′18″E). Additionally, for transcriptome RNA extraction, tender shoots, young leaves, current-year branches, and immature fruits were collected from the same adult plant. The transcriptome samples were immediately frozen in liquid nitrogen after collection and subsequently stored at −80 °C. DNA and RNA extraction and sequencing were performed by Wuhan Benagen Technology Co. Ltd. in Wuhan, China.

### Genome sequencing

A modified CTAB method was performed to extract total DNA from young *C. chago* leaves^11^. The concentration of DNA was assessed using NanoDrop (NanoDrop Technologies, Wilmington, DE, USA) and a Qubit 3.0 fluorometer (Life Technologies, Carlsbad, CA, USA). The purity and integrity of the resulting DNA were assessed using 1% agarose gel electrophoresis. The short-read library with a DNA-fragment insert size of 200–400 bp was prepared using 1 μg genomic DNA following the manufacturer’s instructions (BGI) and was subjected to paired-end (PE) sequencing on a DNBSEQ-T7 platform (BGI Inc., Shenzhen, China) using a PE 150 model, which consequently produced 58.83 Gb (~ 196 M reads, approximately 50×) of raw data (Supplementary Table S2).

Genomic DNA was purified using a DNeasy Plant Mini Kit before HiFi sequencing (Qiagen, Germantown, MD, USA), and its integrity was assessed using a Femto Pulse instrument (Agilent Technologies, Santa Clara, CA, USA). Subsequently, Megaruptor 3 (Diagenode SA., Seraing, Belgium) was employed to fragment 8 μg of genomic DNA, and the resulting fragments were concentrated using AMPure PB magnetic beads (Pacific Biosciences, Menlo Park, CA, USA). Each PacBio single molecule real-time (SMRT) library was constructed using a SMRT bell express template prep Kit 3.0 (Pacific Biosciences, Menlo Park, CA, USA), with insert sizes of 30 kb selected via the BluePippin system (Sage Science, Beverly, MA, USA). The library was then sequenced on a Pacific Bioscience Revio platform in CCS mode, and the raw data were processed into high-fidelity (HiFi) reads using the CCS workflow 7.0.0^12^ with parameters (–streamed–log-level INFO–stderr-json-log–kestrel-files-layout–min-rq 0.9–non-hifi-prefix fail–knrt-ada–pbdc-model). This process yielded approximately 91.73 Gb (~ 80×) of HiFi data with an average read length of about 18 kb and an N50 read length of approximately 18 kb (Supplementary Table S3).

The Nanopore DNA library was prepared using SQK-LSK109 Kit (Oxford Nanopore Technologies, Oxford, UK), and the library was sequenced using a Nanopore PromethION sequencer. Totally about 33.27 Gb (~ 30 x) WGS ONT data were obtained (Supplementary Table S3).

### Hi-C library construction and sequencing

Fresh leaf tissue was fixed in formaldehyde solution, and the cross-linked DNA was then digested and labelled with Biotin. Subsequently, the DNA fragments were ligated together using DNA ligase, then the ligated DNAs were then uncross linked, sheared, and purified. After adding A-tailing and an adapter to the DNA fragments, the biotin-labelled fragments were then enriched using streptavidin magnetic beads. The Hi-C libraries were PCR-amplified and then sequenced on the Illumina NovaSeq 6000 platform in PE150 mode (Supplementary Table S4).

### Transcriptome sequencing

Total RNA from leaves, stems, fruits, and roots of the same plant was isolated. For NGS RNA-Seq, libraries were prepared using the VAHTS Universal V6 RNA-seq Library Prep Kit for Illumina. The libraries were then sequenced on the Illumina NovaSeq 6000 S4 platform. For Full-length isoform sequencing (Iso-Seq), both SQK-PCS109 and SQK-PBK004 Kits (Oxford Nanopore Technologies, Oxford, UK) were used to prepare the library, and the library was sequenced using a Nanopore PromethION sequencer. Finally, a total of 16 Gb (~ 109 M reads) NGS RNA-Seq data and 19 Gb (~ 17 M reads) full-length Iso-Seq data were obtained for genome annotation (Supplementary Tables S5, S6, S7).

### Genome size estimation

Both flow cytometry (FCM) analysis and k-mer frequency analysis were employed to estimate the genome size of *C. chago*. For FCM analysis, the DNA content was assessed using the BD FACScalibur (BD Biosciences, USA), with maize B73 as reference standards. The frequencies of 19-mers, 25-mers, 29-mers, 39-mers and 49-mers were estimated with the software GCE v1.0.0^13^ using HIFI reads. The estimated genome size was ~1.1 Gb, with a genome heterozygosity of 0.8% (Supplementary Table S8).

### Chromosome-level genome assembly

PacBio HiFi reads, WGS ONT reads, and Hi-C reads were assembled into contigs using Hifiasm v0.19.5-r592^14^. The primary assembly was selected for subsequent analysis. Hi-C reads were aligned to the reference genome using Juicer 3, followed by initial HiC-assisted chromosome assembly using 3D-DNA v180922^15^ (with the parameters–early-exit -m haploid -r 0). Manual inspection and adjustment were performed using Juicebox v1.11.08^16^ (pre -n -q 0 or 1), primarily focusing on refining chromosome segment boundaries and correcting assembly errors. Chromosome scaffolding was then performed separately for each chromosome using 3D-DNA, followed by manual adjustments in Juicebox, including removal of erroneous insertions and orientation adjustments, aiming to correct visible errors as much as possible. After manual inspection, the final genome assembly consisted of 12 chromosomes and un-anchored sequences. Gaps with a fixed length of 100 bp were present; therefore, gap filling was performed using quarTeT v1.1.2^17^ software based on HiFi reads.

Most chromosomal telomeres exhibited telomeric repeat sequences (TTTAGGG)n^18^; however, there were individual cases where this sequence was shorter or absent, suggesting incomplete assembly or insufficient extension. To address this, the HiFi reads were mapped back to the chromosomes, and reads mapping near the telomeres were selected. These reads were then assembled into contigs using Hifiasm v0.19.5-r592. The contigs were mapped to the chromosomes, and the chromosomes were extended outward to assemble the telomere sequences as completely as possible. GetOrganelle v1.7.5^19^ was used to assemble the chloroplast and mitochondrial genomes.

The assembly were polished using Nextpolish2 v0.1.0^20^ based on HiFi and NGS short reads. Then, redundancies including rDNA fragments and haplotigs were removed using Redundans v0.13c^21^ (with the parameters -identity 0.98 -overlap 0.8) with manual curation. About 99.92% of the assembled data was anchored to the 12 pseudochromosomes, and the chromosomes were numbered according to the published genome assembly of *C. kanehirae*^22^; 0.07%, and 0.01% of the assembled data was the mitochondrial and chloroplast genomes, respectively (Table 1; Fig. 1b,c; Supplementary Table S1). Finally, we obtained a high-quality genome of *C. chago*.

### Identification of repetitive elements

EDTA v1.9.9^23^ was utilized for de novo identification of transposable elements (parameters:–sensitive 1–anno 1) to generate a TE library. RepeatMasker v4.0.7^24^ (with the parameters -no_is -xsmall) was then employed to identify repetitive regions in the genome. A total of 1,366,885 repetitive sequences were identified, comprising a cumulative length of 676,297,749 bp, accounting for 63.73% of the genome. Among these, the most abundant were LTR elements, with a total of 466,655 elements spanning 431,972,996 bp, making up 40.71% of the genome (Supplementary Table S9).

### Gene identification and functional annotation

Homologous protein evidence was prepared by merging a total of 507,642 non-redundant protein sequences sourced from publicly available proteins for gene annotation, including *Amborella trichopoda*^25^, *Nymphaea colorata*^26^, *Aristolochia fimbriata*^27^, *Piper nigrum*^28^, *Saururus chinensis*^29^, *Annona glabra*^30^, *Liriodendron chinense*^31^, *Magnolia sinica*^32^, *Chimonanthus salicifolius*^33^, *Cinnamomum kanehirae*^22^, *Cinnamomum camphora*^34^, *Litsea cubeba*^35^, *Lindera megaphylla*^36^, *Chloranthus sessilifolius*^37^, *Acorus gramineus*^38^, *Oryza sativa*^39^, *Tetracentron sinense*^40^, and *Arabidopsis thaliana*^41^.

Transcript evidence preparation involved two approaches for NGS transcriptome data: 1) Trinity v2.0.6^42^ was employed to perform *de novo* assembly, and 2) hisat2 v2.1.0^43^ was utilized to map reads to the genome, followed by assembly using StringTie v2.1.5^44^. For iso-seq data, Minimap2 v2.24^45^ (with the parameters -a -x splice–end-seed-pen = 60–G 200k) was used to map reads to the genome, which were subsequently assembled using StringTie v2.1.5 (with the parameters -L -t -f 0.05) (Supplementary Table S10). Gene structure annotation was performed, by employing the PASA (Program to Assemble Spliced Alignments) pipeline v2.4.1^46^ based on the transcript evidence obtained, and full-length genes were identified through comparison with reference proteins. To optimize gene prediction, AUGUSTUS v3.4.0^47^ was trained using the full-length gene set, undergoing five rounds of optimization. Additionally, SNAP^48^ was also trained to further enhance gene prediction accuracy.

The MAKER2 v2.31.9^49^ annotation workflow was employed to annotate genes based on *ab initio* prediction, transcript evidence, and homologous protein evidence. In this step, repetitive regions were first masked using RepeatMasker v4.0.7. AUGUSTUS v3.4.0 and SNAP were used for *ab initio* gene prediction. Then, the assembled transcript sequences were aligned with the genome using BLASTN, while protein sequences were aligned using BLASTX, and the alignments were optimized using Exonerate v2.2.0^50^. Hints files were generated based on the evidence obtained, which were then integrated with AUGUSTUS and SNAP to predict gene models.

Further integration of MAKER and PASA annotations was performed using EVidenceModeler (EVM) v1.1.1^51^ to generate consistent gene annotations. TEsorter v1.4.1^52^ was utilized to identify TE protein domains in the genome, which were subsequently masked by EVM v1.1.1, to avoid introducing transposable element (TE) coding regions. Finally, PASA v2.4.1 was used to upgrade and optimize the results obtained by EVM, add UTRs, and add alternative splicing. Gene annotations with abnormal coding frames and those that were too short (<50 aa) were removed. Barrnap v0.9 (https://github.com/tseemann/barrnap) and tRNAScan-SE v1.3.1^53^ were used to annotate rRNA and tRNAs respectively. Various non-coding ncRNAs were annotated using RfamScan v14.2^54^.

Functional annotation of protein-coding genes was conducted using three strategies. 1) the predicted genes were aligned with the eggNOG v. 5.0 homologous gene database using eggNOG-mapper v. 2.0.0^55^ (–target_taxa Viridiplantae -m diamond) for Gene Ontology (GO) and Kyoto Encyclopedia of Genes and Genomes (KEEG) annotation. 2) sequence matching was performed using DIAMOND v0.9.24^56^ (–evalue 1e-5–max-target-seqs 5) (Identity >30%, E-value <1e-5), aligning the protein sequences with various databases such as Swiss_Prot, TrEMBL, NR (non-redundant protein), and Arabidopsis, to identify best gene matches. 3) InterProScan v5.27-66.0^57^ was used to obtain the conserved amino acid sequences, motifs, and domains of the predicted proteins by searching for similarity of domain according to the sub-databases PRINTS, Pfam, SMART, PANTHER and CDD of the InterPro database (Table 3). Finally, 27,795 genes were functionally annotated in at least one of the above databases, accounting for 96.91% of the predicted protein-coding genes (Table 2; Supplementary Table S11).

**Table 3:** Statistics of functional annotation result of *Cinnamomum chago* genome.

Program	Database	Number	Percent (%)
eggNOG-mapper	GO	12845	44.79%
	KEGG_KO	12343	43.04%
	EC	5428	18.93%
	KEGG_Pathway	7713	26.89%
	eggNOG	24520	85.49%
	COG	26212	91.39%
DIAMOND	Swiss_Prot	20334	70.90%
	TrEMBL	27261	95.05%
	NR	26136	91.13%
	TAIR10	23921	83.40%
InterProScan	CDD	8999	31.38%
	Pfam	22499	78.45%
	SUPERFAMILY	17369	60.56%
	Interpro	23514	81.98%
	Coils	4396	15.33%
	Gene3D	18690	65.17%
	Phobius	9861	34.38%
	PRINTS	3970	13.84%
	TIGRFAM	2897	10.10%
	SMART	8176	28.51%

Mitochondrial and chloroplast genomes were also annotated using OGAP pipeline (https://github.com/zhangrengang/ogap). Totally, 61 genes and 108 genes were functionally annotated in mitochondrial and chloroplast genomes, respectively (Supplementary Table S12).

## Data Records

The relevant data reported in this paper have been deposited in the National Genomics Data Center, Beijing Institute of Genomics, Chinese Academy of Sciences/China National Center for Bioinformation, under the BioProject accession number PRJCA022354 that is publicly accessible at https://ngdc.cncb.ac.cn/gwh. BGI short-reads, PacBio HiFi long-reads, Hi-C reads, WGS ONT data, Iso-Seq data and RNA-Seq data have been deposited in the Genome Sequence Archive (GSA) in NGDC under the accession number CRR1001223^58^, CRR1001224^59^, CRR1001225^60^, CRR1091096^61^, CRR1091097^62^ and CRR1001228^63^. The final chromosome assembly and annotation data were deposited in the Genome Warehouse (GWH) in NGDC under the accession number GWHERBI00000000^64^. GSA and GWH data are also available in NCBI SRA and GenBank under the accession number SRR27371173^65^, SRR27371174^66^, SRR27371175^67^, SRR27371176^68^, SRR28466993^69^, SRR28466994^70^, and GCA_038049695.1^71^. Annotation data are available in Figshare^72^.

## Technical Validation

### Genome assembly quality assessment

The final assembly was about 1.1 Gb, similar with the results from K-mer analysis (Supplementary Table S8; Supplementary Figure S1). There was only one gap in the assembly, contig N50 reached 92.10 Mb, which showed good continuity of the assembly. Short reads were mapped to the genome using BWA-MEM v0.7.17-r1188^73^, while the third-generation reads were mapped using Minimap2 v2.24^45^. Non-primary alignments were filtered out, and the mapping ratio and coverage percentage were calculated. The results are shown in Table 4, indicating a high level of sequence coverage for the genome. According to BUSCO (Benchmarking Universal Single-Copy Orthologs) v5.3.2^74^, the proportion of complete core genes (including single-copy and duplicated genes) was found to be 99.0%. The percentage of missing genes was 0.5%, indicating a high level of gene completeness.

**Table 4:** Mapping ratio and coverage percentage of different data sets.

Data set	Reads mapped	Bases mapped	>=1×	>=5×	>=10×	>=20×
HiFi	99.52%	99.53%	100.00%	99.97%	99.94%	99.68%
Iso-Seq	98.05%	99.17%	24.61%	11.82%	7.47%	4.81%
RNA-Seq	92.73%	92.47%	14.24%	7.56%	5.43%	3.96%
Short reads	98.70%	98.70%	99.69%	98.74%	97.25%	92.28%

According to the relationship between guanine-cytosine (GC) distribution and sequencing distribution, there was significant GC bias in short reads but no obvious bias in long reads (Supplementary Figure S2). The Hi-C data was further mapped onto the final genome assembly using Juicer v1.5.6^16^, revealing a well-executed chromosome clustering effect (Supplementary Figure S2) with no apparent chromosomal assembly errors.

The genome assembly quality was also assessed by the LTR assembly index (LAI)^75^, consensus quality (QV)^76^, contig/chromosome ratio (CC ratio)^77^, and Clipping information for Revealing Assembly Quality (CRAQ)^78^. The LAI of the assembled genome was 10.80 (>10), indicating the assembly has reached the level of the reference genome. QV of the assembled genome was approximately 70.12, indicating an accuracy of over 99.99% in the assembly. CC ratio of the assembly was 1.25, which reflects high continuity of the assembly. According to CRAQ, regional and structural assembly quality indicators (R-AQI and S-AQI) were approximately 95.31 and 97.73, respectively, which corresponds to low assembly errors (Supplementary Table S13).

**Fig. 2:**
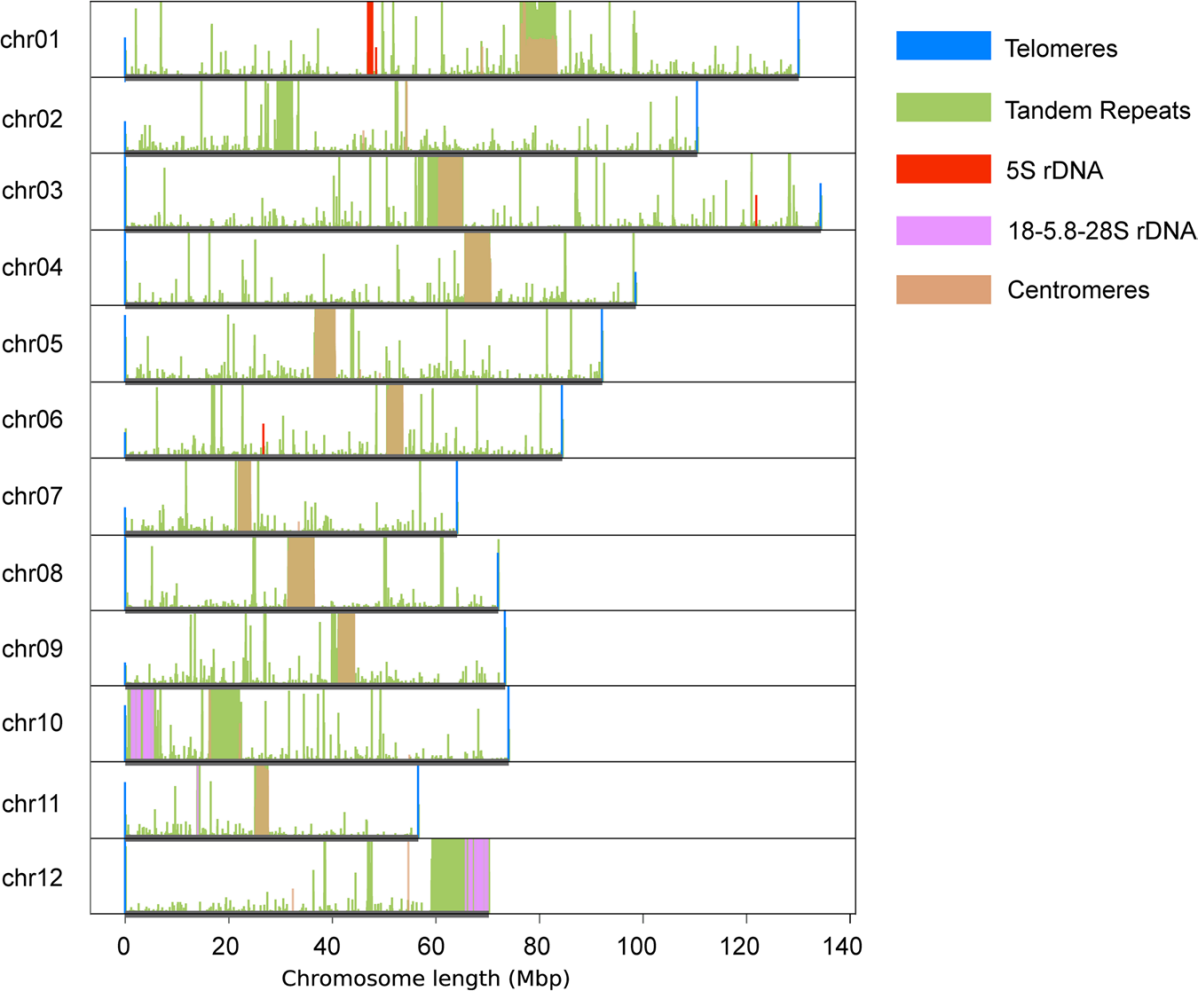
The distribution of repeated elements on the chromosomes: telomeric TTTAGGG, tandem repeat, 5 S rDNA, 18-5.8-28 S rDNA, and putative centromeres. The vertical axis represents the count of repeated elements within 20k intervals.

The repetitive sequences were mapped to the genome to determine the position of the telomeres and other characteristic sequences on the chromosomes. Most of the chromosomes assembled complete telomere sequences (TTTAGGG), and only one telomere was missing. Putative centromere tandem repeat motif (GCGGCTCTAGAAAATTGTTGACTCTACACTGTGTTTCATGCGACTCTTGGTCCAAAGACTCCCTCTAGAAAAATCCGGGATCACGTTTTACTCTAAAAGGGGTTTCGGGTGTCCTTCTCTTGTCTTACGCCTCTAAATCCATTTGAAGGGATTCTGGGTTGAGATGCGCTTTTTAGGATATTTCGAGCTACTTTTCGGTTTAAAACGGGTTTCGGGTGAATCTTGGGTATGGAAAACACTTTCGGGGAGTTCAGTGTTTGTAAAGGCGAAAACCCGAACTTCGTGCGGGTCGTACGGTACTTTTGTACGAAAACACAATCTAT) was identified from HiFi reads using Centromics (https://github.com/zhangrengang/Centromics). Most chromosomes contained the large tandem repeat regions as putative centromere (Fig. 2). In addition, the 18-5.8-28 S rDNA arrays were detected on three chromosomes including Chr10, Chr 11 and Chr12, while 5 S rDNA arrays were found on Chr01, Chr03 and Chr06 (Fig. 2). In summary, this assembly can by described as a nearly telomere-to-telomere genome.

### Evaluation of the gene annotation

The integrated and annotated proteins were evaluated using BUSCO with the lineage dataset embryophyta_odb10. Among a total of 1614 BUSCO groups, 98.6% BUSCO groups were fully covered (including 52.1% single-copy genes and 46.5% duplicated genes), 0.3% groups were fragmented and 1.1% were missing, which showed high quality annotation of the annotation (Table 5).

**Table 5:** BUSCO assessment result.

Type	Number
Complete BUSCOs (C)	1,598 (99.0%)
Complete and single-copy BUSCOs (S)	1,536 (95.2%)
Complete and duplicated BUSCOs (D)	62 (3.8%)
Fragmented BUSCOs (F)	8 (0.5%)
Missing BUSCOs (M)	8 (0.5%)
Total BUSCO groups searched	1,614

### Supplementary information

Supplementary Figures

Supplementary Tables

## Data Availability

All commands and pipelines used were performed according to the manuals or protocols of the tools used in this study. The software and tools used are publicly accessible, with the version and parameters specified in the Methods section. If no detailed parameters were mentioned, default parameters were used. No custom code was used in this study.
