# Supplementary material for: Chromosome-level genome assembly of the threatened resource plant Cinnamomum chago
Source: Sci Data. 2024 May 3;11:447. doi: 10.1038/s41597-024-03293-1 (PMC11068913; doi:10.1038/s41597-024-03293-1)
Supplement: Supplementary file 1 — Supplementary Figures [file 41597_2024_3293_MOESM1_ESM.pdf]

Supplementary Figures S1–S2

Supplementary Figure S1. The depth-distribution of K-mers.

Supplementary Figure S2. The distribution of coverage depth of the genome (left) and BUSCO core region (middle), and coverage depth distribution (right) evaluated by the next-generation data (upper) and HiFi data (lower).

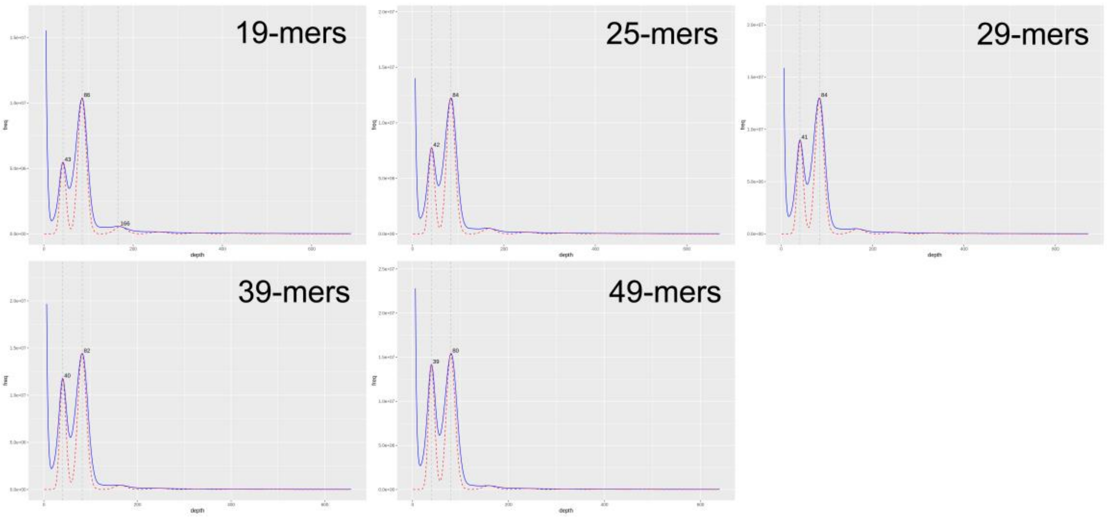

Supplementary Figure S1. The depth-distribution of K-mers.

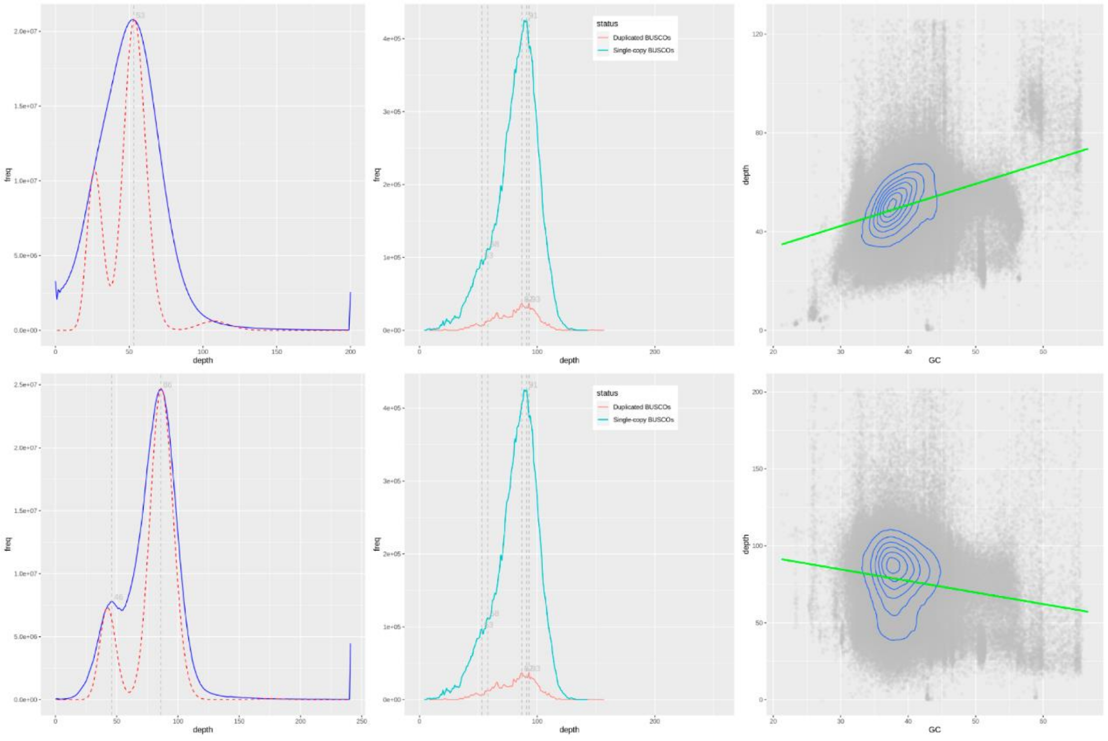

Supplementary Figure S2. The distribution of coverage depth of the genome (left) and BUSCO core region (middle), and coverage depth distribution (right) evaluated by the next-generation data (upper) and HiFi data (lower).
